# Implementing precision oncology for sarcoma patients: the CCC^LMU^molecular tumor board experience

**DOI:** 10.1007/s00432-023-05179-y

**Published:** 2023-08-05

**Authors:** Luc M. Berclaz, Anton Burkhard-Meier, Philipp Lange, Dorit Di Gioia, Michael Schmidt, Thomas Knösel, Frederick Klauschen, Michael von Bergwelt-Baildon, Volker Heinemann, Philipp A. Greif, C. Benedikt Westphalen, Kathrin Heinrich, Lars H. Lindner

**Affiliations:** 1 Department of Medicine III, University Hospital, Ludwig-Maximilians-University (LMU) Munich, Marchioninistr. 15, 81377 Munich, Germany; 2 Department of Psychology, Philipps-Universität Marburg, Marburg, Germany; 3 Munich Cancer Registry, Institute of Medical Information Processing, Biometry and Epidemiology, Ludwig-Maximilians-University (LMU) Munich, Marchioninistr. 15, 81377 Munich, Germany; 4 Institute of Pathology, Ludwig-Maximilians-University (LMU) Munich, Munich, Germany; 5 Comprehensive Cancer Center Munich and Department of Medicine III, University Hospital, Ludwig-Maximilians-University (LMU) Munich, Marchioninistr. 15, 81377 Munich, Germany; 6 German Cancer Consortium (DKTK), partner site Munich, 81377 Munich, Germany; 7 German Cancer Research Center (DKFZ), 69121 Heidelberg, Germany

**Keywords:** Sarcoma, Next-generation sequencing, Molecular tumor board, Precision oncology

## Abstract

**Purpose:**

Due to poor outcomes and limited treatment options, patients with advanced bone and soft tissue sarcomas (BS/STS) may undergo comprehensive molecular profiling of tumor samples to identify possible therapeutic targets. The aim of this study was to determine the impact of routine molecular profiling in the setting of a dedicated precision oncology program in patients with BS/STS in a German large-volume sarcoma center.

**Methods:**

92 BS/STS patients who received comprehensive genomic profiling (CGP) and were subsequently discussed in our molecular tumor board (MTB) between 2016 and 2022 were included. Patient records were retrospectively reviewed, and the clinical impact of NGS-related findings was analyzed.

**Results:**

89.1% of patients had received at least one treatment line before NGS testing. At least one molecular alteration was found in 71 patients (82.6%). The most common alterations were mutations in *TP53* (23.3% of patients), followed by *PIK3CA* and *MDM2* mutations (9.3% each). Druggable alterations were identified, and treatment recommended in 32 patients (37.2%). Of those patients with actionable alterations, ten patients (31.2%) received personalized treatment and six patients did benefit from molecular-based therapy in terms of a progression-free survival ratio (PFSr) > 1.3.

**Conclusion:**

Our single-center experience shows an increasing uptake of next-generation sequencing (NGS) and highlights current challenges of implementing precision oncology in the management of patients with BS/STS. A relevant number of patients were diagnosed with clinically actionable alterations. Our results highlight the potential benefit of NGS in patients with rare cancers and currently limited therapeutic options.

## Introduction

Bone and soft tissue sarcomas (BS/STS) represent a heterogeneous group of mesenchymal malignancies. They account for approximately 1% of adult and 15% of pediatric malignancies (Beckingsale and Shaw 2017). Overall prognosis is poor, and up to 50% of sarcoma patients develop metastatic disease (Italiano et al. 2011; Marko et al. 2016). With survival rates around 12–24 months under treatment with standard chemotherapy, prognosis remains dismal in advanced and metastatic stages (Van Glabbeke et al. 1999; Lochner et al. 2020). In the context of systemic therapy, there has only been little progress in the treatment of BS/STS in the past 3 decades. In STS, despite the current knowledge about clinical and biological differences, patients have been treated in a “one-size-fits-all” approach with anthracyclines and alkylating agents (Katz et al. 2018). Histology-tailored chemotherapy regimens have not led to improved outcomes over the standard first-line chemotherapy regimen in STS (Gronchi et al. 2020). The only FDA-approved first-line targeted therapy in STS represents tyrosine kinase inhibitors (TKI) including imatinib for gastrointestinal stroma tumors (GIST) and dermatofibrosarcoma protuberans (DeMatteo et al. 2009). Systemic therapy for BS includes conventional chemotherapeutic agents as part of subtype specific protocols (Ferrari et al. 2018; Brennan et al. 2020; Smeland et al. 2019). Drug resistance is believed to cause treatment failure in over 90% of patients with metastatic cancer, which underlines the need for additional therapy lines and novel therapeutic approaches in patients with BS/STS (Longley and Johnston 2005).

Next-generation sequencing (NGS) enables parallel sequencing of RNA and DNA from (archived) tumor tissue. It has affected diagnostic and therapeutic management in many advanced malignancies and has been increasingly used for focused biomarker screening in colorectal cancer, non-small-cell lung cancer (NSCLC), and breast cancer (Van Cutsem et al. 2016; Sosman et al. 2012; Gennari et al. 2021; Planchard et al. 2018; Chakravarty et al. 2022). Taking into account the frequency of actionable alterations and a potential therapeutic benefit, the European Society of Medical Oncology (ESMO) recommends the routine use of multigene NGS in advanced NSCLC, prostate cancer, ovarian cancer, and cholangiocarcinoma in addition to tumor-specific molecular targets (Mosele et al. 2020). Importantly, there are now six drugs FDA-approved in a histology–agnostic fashion for a total of six different biomarkers, underscoring the need for scaling of tumor profiling within sustainable structures.

In BS/STS, the role of NGS in routine clinical practice is not yet defined. Implementation of molecular tumor boards (MTB) for interdisciplinary discussion is a valid option to integrate NGS results into clinical routine. At the CCC^LMU^ Munich, a precision oncology program was initiated in 2016. Heinrich et al. demonstrated feasibility of the program in a retrospective analysis of the first 1000 patient contacts of the program. In this single-center analysis, 41% of patients received a treatment recommendation based on comprehensive genomic profiling (CGP). Despite the high rate of treatment recommendations, only 17% of all patients received treatment based on MTB discussion, being in line with previously published reports (Heinrich et al. 2022; Tannock and Hickman 2019).

Here, we aim to determine the feasibility and impact of routine NGS and molecular tumor boards in patients with BS/STS treated in large German Sarcoma referral center. Our results indicate that a relevant proportion of patients with BS/STS harbor druggable alterations, but only a fraction ultimately receive recommended treatments. This study highlights current challenges in the clinical implementation of comprehensive genomic profiling in BS/STS.

## Materials and methods

### Patient selection

Eligible patients had pathologically confirmed bone or soft tissue sarcoma (BS/STS). Treatment options were discussed in a multidisciplinary team within the LMU Munich Sarcoma Center (SarKUM) between August 2016 and August 2022. If at least one of the criteria for comprehensive genomic profiling (young age, rare histopathological subtype, and limited therapeutic options) was met, patients were presented to the CCC^LMU^ Munich Precision Oncology program after discussion within the sarcoma tumor board. Clinical, pathological, and outcomes data were extracted from our MTB database.

### NGS-based genomic profiling

Comprehensive genomic profiling (CGP) via targeted NGS was performed at the LMU Munich Institute of Pathology. Patients with externally performed CGP were registered and discussed within our MTB in select cases. Starting in 2016, in-house panel sequencing was performed with Oncomine™ Focus Assay, a 52-gene panel, which was continuously replaced by the 161-gene panel Oncomine™ Comprehensive Assay (﻿Thermo Fisher, Darmstadt, Germany) and the TruSight Oncology (TSO) 500™ Assay (Illumina, San Diego, USA). All panels allow simultaneous RNA and DNA sequencing with detection of insertions/deletions (indels), gene fusions, single-nucleotide variants (SNV), and copy number variations (CNV). Additionally, tumor mutational burden (TMB) was evaluated in select cases (Oncomine™ Tumor Mutational Load Assay, ThermoFisher Scientific). CGP was either performed on FFPE tumor tissue or liquid biopsies (e.g., cerebrospinal fluid, blood, or ascites). Material from primary tumor, metastases, or locally recurrent tumor was used. Young patients with rare sarcomas were referred to the National Center for Tumor Diseases (NCT) and German Cancer Consortium (DKTK) Molecularly Aided Stratification for Tumor Eradication (MASTER) program for whole genome/exome and transcriptome analysis in select cases (Horak et al. 2017, 2021).

### Interpretation of molecular tumor board recommendations

Following molecular testing, results were individually discussed in a dedicated molecular tumor board (MTB) consisting of pathologists, medical oncologists, geneticists, and representatives from organ-specific tumor boards. To define clinical actionability of molecular alterations, a structured database query was performed. In addition, an on-site literature database was created within the LMU Munich MTB. For this study, druggable alterations were assigned to baskets based on the cellular pathway involved according to a modified classification by Horak et al. (2021): Tyrosine kinases (TK), *PI3K-AKT-mTOR* (PAM), DNA damage repair (DDR), *RAF-MEK-ERK* (RME), Cell cycle (CC), Immune evasion (IE), and *IDH* mutations (IDH). Further druggable alterations not associated with one of these cellular pathways were termed Other (OTH). After discussion of NGS results in our MTB, clinical implementation of MTB recommendations was discussed in a multidisciplinary team within the LMU Munich Sarcoma Center (SarKUM).

### Statistical analysis

Descriptive and statistical analyses, as well as the generation of graphs were performed with IBM SPSS Statistics Version 28.0, R Version 4.2.2 and Microsoft 365 Version 2206. The comparison of the mean age of two groups was performed using the Student’s *t* test. Survival time was calculated from the initial diagnosis to either death or the date of last contact. Statistical significance was determined as a *p* value < 0.05. Linear regression was computed to calculate the association between date of registration and the number of days to MTB discussion. To measure the clinical benefit of MTB-based therapies, the progression-free survival ratio (PFSr, PFS under MTB-based therapy divided by PFS of prior systemic therapy) was calculated (Von Hoff et al. 2010). The chosen cut-off to measure a therapeutic effect was set to > 1.3 according to previous studies (Massard et al. 2017; Horak et al. 2021).

## Results

### Patient characteristics and molecular tumor board workflow

**Fig. 1:**
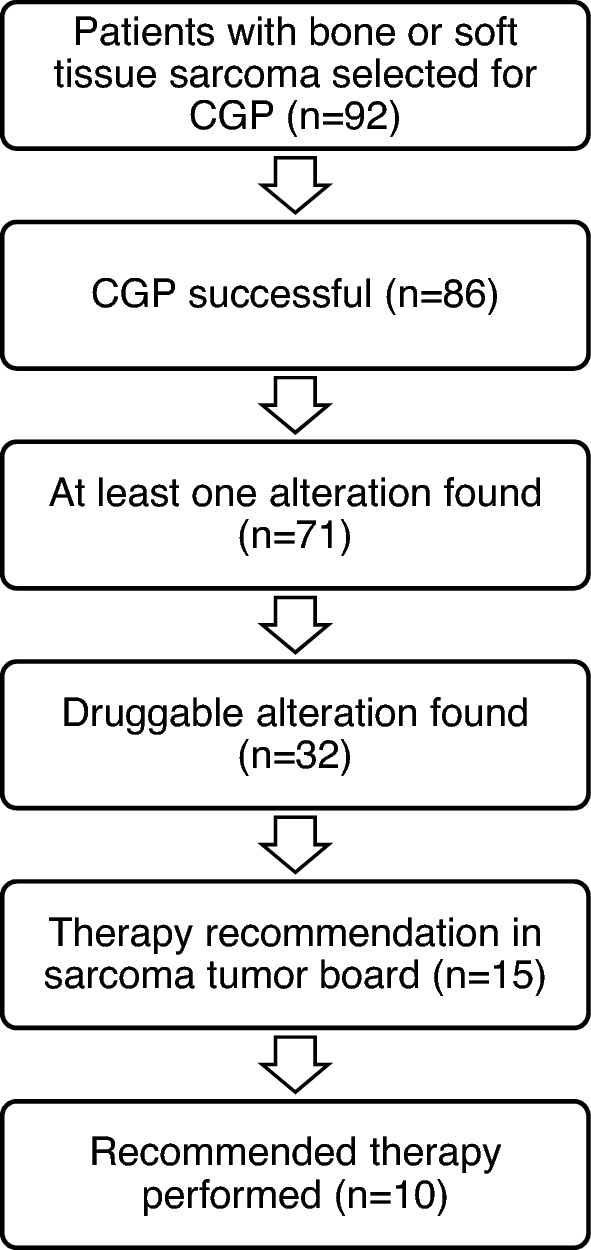
Molecular tumor board workflow in patients with bone and soft tissue sarcomas. *CGP* comprehensive genomic profiling

**Fig. 2:**
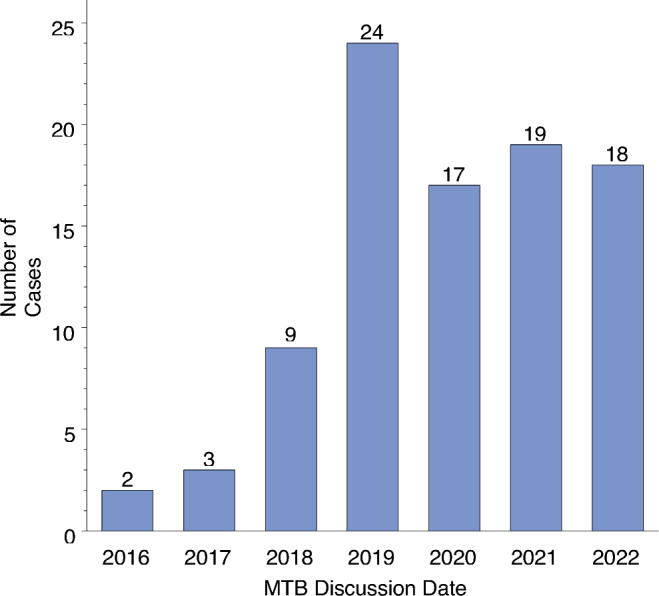
MTB discussions over the examined study period

The clinicopathologic characteristics of the study cohort are summarized in Table 1. In total, 92 patients with histologically confirmed bone or soft tissue sarcoma (BS/STS) and presentation at our molecular tumor board (MTB, *n = *89) and/or the DKTK MASTER program (*n = *5) between August 2016 and August 2022 were analyzed (Fig. 1). Median age at initial diagnosis was 49 years (range 0–80 years), and 52.2% of patients were female. The median time interval between initial diagnosis and MTB discussion was 18.7 months (range 0.3–283.2 months). For patients with synchronous metastatic disease, this time interval was 7.6 months (range 0.3–85.8 months). Most common histological subtypes were liposarcoma (*n = *11, 12%), undifferentiated pleomorphic sarcoma (*n = *11, 12%), leiomyosarcoma (*n = *9, 9.8%) and chondrosarcoma (*n = *9, 9.8%). 84 sarcomas (91.4%) were classified as high grade. Metastatic disease was present in 43.5% of patients at initial diagnosis and in 84.8% of patients at date of CGP. Costs for NGS testing were covered by health insurance providers in all patients. While the number of sequenced and discussed patients increased over time (Fig. 2), turnaround time between CGP and MTB discussion decreased over the examined period (median 62.5 days in 2016/2017 vs. 33 days in 2021, *β = *− 6.153, *p = *0.20). After exclusion of two external patients with high turnaround times, the effect became significant (*β = *− 5.512, *p = *0.014). 82 patients (89.1%) underwent at least one local or systemic treatment before discussion of CGP results, with a median number of two previous systemic (range 0–6) and two local (range 0–10) therapies (Table 2). MTB referral was initiated by our medical oncology department in 75.8% of cases.

**Table 1:** Baseline characteristics

Factor	Strata	*N*	%
Total		92	100
Sex	Male	44	47,8
	Female	48	52.2
Health insurance status	Public/statutory	64	69.6
	Private	28	30.4
Histological subtype	All STS	**72**	**78.3**
	Liposarcoma	11	12
	UPS	11	12
	Leiomyosarcoma	9	9.8
	Synovial sarcoma	7	7.6
	Angiosarcoma	5	5.4
	(Myxo-)Fibrosarcoma	5	5.4
	Rhabdomyosarcoma	4	4.3
	Uterine sarcoma (non-LMS)	4	4.3
	GIST	3	3.3
	Other	13	14.1
Histological subtype	All BS	**20**	**21.7**
	Chondrosarcoma	9	9.8
	Osteosarcoma	7	7.6
	Ewing sarcoma	4	4.3
Localization	Extremities	42	45.6
	Abdominal	28	30.4
	Trunk	17	18.5
	Head/Neck	3	3.3
	Other	2	2.2
Tumor grading	High	84	91.4
	Low	8	8.6
Presence of metastases at initial diagnosis	Yes	40	43.5
	No	52	56.5
Presence of metastases at MTB discussion	Yes	78	85
	No	14	15
Used tumor material for genomic analysis	Primary tumor	46	50
	Metastasis	33	35.9
	Locally recurrent tumor	12	13.1
	Cerebrospinal fluid	1	1
	Blood	1	1
Status at last follow-up	Alive	54	58.7
	Deceased	38	41.3

**Table 2:** List of patients with therapeutic change after MTB discussion

Patient	Histological subtype	Actionable alteration	MTB-based therapy	Therapy Line	PFS (in months)	PFSr
1	Chondrosarcoma	IDH1	Ivosidenib	2	NR (≥ 33)	≥ 1.8
2	Chondrosarcoma	IDH1	Ivosidenib	4	7	3.5
3	Ewing sarcoma	BRCA2	Olaparib^a^ (+ Temozolomid + Irino-tecan)	3	17	1.9
4	Osteosarcoma	ALK	Crizotinib^b^	4	3	0.4
5	Angiosarcoma	PDGFRA, KIT	Pazopanib	1	3	–
6	Angiosarcoma	ARID1A	Pembrolizumab^a^ (+ Paclitaxel)	3	3	0.3
7	Myxofibrosarcoma	ERBB2	Trastuzumab/Pertuzu-mab^a^ (+ Gemcitabine)	5	14	4.7
8	Myxoid Liposarcoma	PIK3CA	Alpelisib	6	NR (≥ 5)	≥ 2.5
9	Dediff. Liposarcoma	CDK6	Palbociclib	6	6	2
10	GIST	PDGFRA	Imatinib	1	NR (≥ 4)	-

*PFS* Progression-free survival associated with the MTB-based therapy in months, *PFSr* PFS under MTB-based therapy divided by PFS of prior systemic therapy, cut-off for therapy response > 1.3, *NR*  not reached

^a^Patients with combination therapies including chemotherapeutic agents

^b^Therapeutic change based on a positive DKTK MASTER report

CGP assays were performed as previously described (Heinrich et al. 2022). In most patients, FFPE tumor tissue was used for comprehensive genomic profiling. Testing by liquid biopsy was only done in two patients (2.2%). For whole exome/genome and transcriptome sequencing within the DKTK MASTER program, new biopsies were performed to obtain fresh frozen tumor samples for five patients (5.4%). NGS analysis was successful in 93.5% of patients, and insufficient quality of tumor material was the main reason for unsuccessful testing. One patient was discussed twice due to additional CGP to detect further alterations. The performed CGP and MTB workflow can be seen in Fig. 1.

Other histological subtypes include 2 intimal sarcomas, 1 sclerosing epithelioid fibrosarcoma, 1 epithelioid sarcoma, 2 clear cell sarcomas, 2 solitary fibrous tumors, 1 phosphaturic mesenchymal tumor, 2 CIC-rearranged sarcomas, 1 alveolar soft part sarcoma, 1 adenosarcoma

### Molecular alterations and actionability

**Fig. 3:**
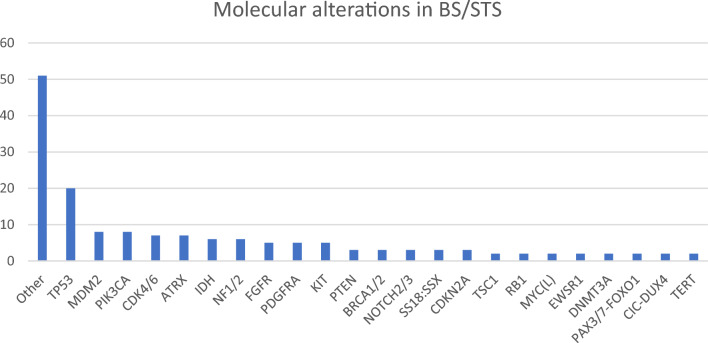
Molecular alterations in BS/STS. Other: rare alterations identified only once in our study collective

**Fig. 4:**
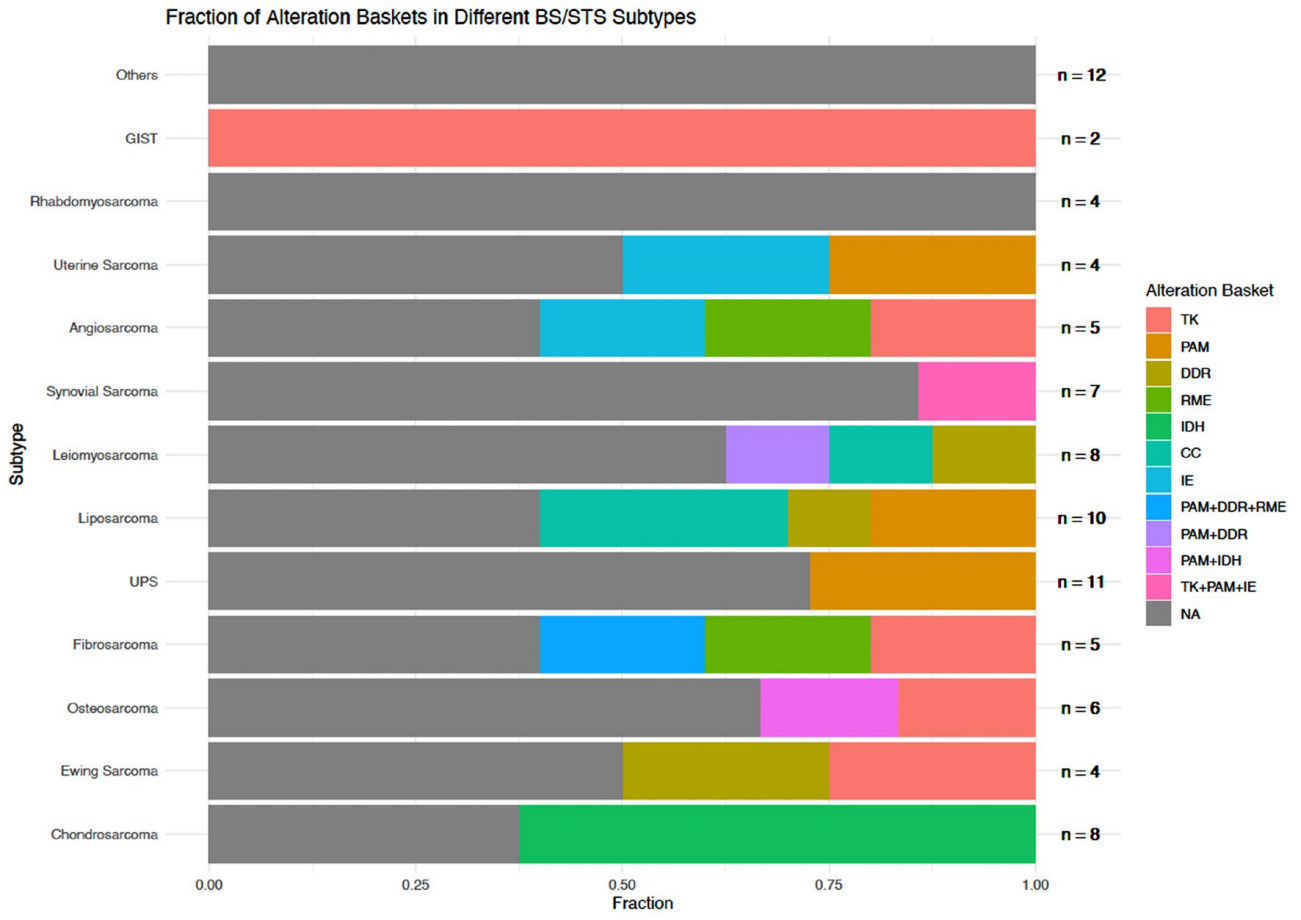
Proportion of actionable alterations in BS/STS subtypes divided into alteration baskets. *TK* tyrosine kinases, *PAM* PI3K-AKT-mTOR, *DDR* DNA damage repair, *RME* RAF-MEK-ERK, *IDH* isocitrate dehydrogenase, *CC* Cell cycle, *IE* immune evasion, *NA* no actionable alterations

CGP was technically successful in 86 of 92 patients (93.5%), and molecular alterations were detected in 71 patients (82.6%). In 32 patients (37.2%), at least one druggable alteration was found. The most common alterations were mutations in *TP53* (*n = *20; 23.3%), followed by *PIK3CA* and *MDM2* alterations (9.3% each). The most common druggable alterations were mutations in *IDH* (7% of patients) and *CDK4/6* (8.1% of patients) (Figs. 3 and 4). Histological subtypes with no druggable alterations were rhabdomyosarcoma and subtypes termed as “Other” including ultra-rare STS such as clear cell sarcoma or sclerosing epithelioid fibrosarcoma (Stacchiotti et al. 2021). As previously mentioned, druggable alterations were assigned to baskets based on the cellular pathway involved according to a modified Horak classification (Horak et al. 2021). Expected disease-defining gene alterations were detected in several histological subtypes, including *MDM2* and *CDK4* in liposarcoma, *IDH* mutations in chondrosarcoma, and alterations related to tyrosine kinases in GIST (*KIT*, *PDGFR*). Of all detected alterations, 53.5% were associated with a loss and 46.5% with a gain of function. Mutations, amplifications, and fusions accounted for 64.3%, 23.6%, and 12.1% of alterations, respectively. Tumor mutational burden (TMB) was assessed in 48 patients (52.2%), and TMB was high (≥ 10 mutations per megabase) in three patients (6.3%).

### Targeted treatment recommendations

In 32 patients (37.2%), CGP detected an actionable alteration and led to a treatment recommendation in our dedicated molecular tumor board. Based on the MTB recommendation or a positive DKTK MASTER report in two cases, our sarcoma-specific tumor board recommended a targeted therapy in 15 patients (17.4%). This finally resulted in an actual treatment change at disease progression in ten patients (11.6%). The remaining five patients did not receive the recommended therapy due to death, rapid tumor progression (*n = *4), or due to a therapy recommendation for an expected progression not yet occurred. The treatment group included two chondrosarcomas, one Ewing sarcoma, one osteosarcoma, two angiosarcomas, two liposarcomas, one myxofibrosarcoma, and one GIST. Mean age at initial diagnosis was lower (*M = *38 years, SD = 0.05) than in the whole cohort (*M = *53 years, SD = 15.53) (*p = *0.046). In the treatment group, median duration from initial diagnosis to CGP was 13.6 months, and the median turnaround time was 41.5 days. Nine patients were treated with off-label therapies after approval by their respective health insurance. The patient with GIST received an FDA-approved therapy with imatinib after NGS and detection of an uncommon *PDGFRA* exon 12 mutation. Six patients derived clinical benefit with a progression-free survival ratio (PFSr) > 1.3, and one patient who received a first-line MTB-based therapy had a PFS of more than three months (Table 2).

## Discussion

Over the last decades, progress has been slow in the treatment of bone and soft tissue sarcomas (BS/STS). While multigene NGS has led to a better understanding of the genetic landscape of sarcomas and allows better subgrouping, it does not yet impact therapeutic algorithms in daily clinical practice. Except for GIST and dermatofibrosarcoma protuberans, there are no approved therapies based on molecular targets specifically for BS/STS. In this retrospective single-center analysis, we set out to evaluate the use of comprehensive genomic profiling (CGP) to drive molecular tumor board recommendations in patients with BS/STS. Although there are several reports on single-center experiences with molecular tumor boards, the role of NGS in routine clinical practice in BS/STS is not sufficiently defined (Cote et al. 2018; Gusho et al. 2022). Current ESMO guidelines recommend molecular pathology for STS when it is required for diagnosis or when it “may have prognostic and/or predictive relevance” (Gronchi et al. 2021). For BS, ESMO guidelines remain equally vague (Strauss et al. 2021).

In our cohort, 82.6% of patients had at least one molecular alteration. Not surprisingly, *TP53* mutations were found as the most common alterations (23.3% of patients), followed by *PIK3CA* and *MDM2* alterations (9.3% each). These alterations are commonly found in BS/STS and correlate with other single-center experiences on NGS in sarcomas (Cote et al. 2018; Gusho et al. 2022; Lucchesi et al. 2018). Expectedly, most molecular alterations including *TP53* mutations did not have a therapeutic consequence. While *TP53* mutations are frequently found in tumor sequencing, they currently do not carry therapeutic relevance and are often associated with poor prognosis in several tumors including breast cancer, Ewing sarcoma, or colorectal cancer (Andrikopoulou et al. 2021; Huemer et al. 2018; Tirode et al. 2014; Hu et al. 2021). *Isocitrate dehydrogenase* (IDH) mutations were found in more than half of patients with chondrosarcoma, being consistent with previous studies (Amary et al. 2011). The efficacy of IDH inhibitors was analyzed in several studies including a phase I trial demonstrating a median PFS of 5.6 months for advanced chondrosarcoma patients (Tap et al. 2020). Interestingly, no druggable alterations were found in our group of ultra-rare sarcomas, such as sclerosing epithelioid fibrosarcomas, intimal sarcomas, or CIC-rearranged sarcomas. In addition to the low number of actionable alterations in translocation-associated sarcomas (Lucchesi et al. 2018), possible explanations could lie in the commercial panels adopted in our study and lack of clinical trials for these tumor entities, which limits the discovery of potential therapeutic targets. Specific studies including CGP in this ultra-rare group are needed for better diagnosis and therapy.

High tumor mutational burden was identified as a predictive parameter for response to immunotherapy in multiple cancer types (Marabelle et al. 2019; Yarchoan et al. 2017). This led to the FDA approval of the PD-1 inhibitor pembrolizumab for TMB-high (TMB, ≥ 10 mutations per megabase) solid tumors. In our cohort, only three patients (6.3% of assessed patients) met this condition, which is consistent with previous literature on BS/STS (Yarchoan et al. 2019). As only a minority of sarcoma patients qualify as TMB-high, the PEMBROSARC trial suggested the immune-active tumor microenvironment with tertiary lymphoid structures as an alternative predictive parameter of response to immunotherapy (NCT02406781).

37.2% of patients harbored druggable alterations leading to a therapeutic recommendation of the MTB and/or the DKTK MASTER program (*n = *5). This rate of druggable alterations is in line with previous studies on comprehensive tumor profiling in sarcomas. Lucchesi et al. demonstrated at least one targetable alteration in 41% of patients with soft tissue sarcomas (Lucchesi et al. 2018). Gusho et al. discovered clinically actionable alterations in 47.1% of patients with BS/STS, and Cote et al. demonstrated a druggable alteration in around 40% of patients (Gusho et al. 2022; Cote et al. 2018). In the DKTK MASTER cohort, Horak et al. presented a rationale for targeted therapies beyond current guidelines in 86.9% of patients with rare cancers including bone and soft tissue sarcomas and 31.8% were treated accordingly. In our cohort, all patients (*n = *5) included in the DKTK MASTER program received a therapeutic recommendation based on their sequencing results. In the end, only one patient received a molecularly targeted therapy after discussion in this program. Due to the identification of an alternative transcript (ATI) on *ALK*, the patient with osteosarcoma underwent a targeted therapy with the ALK-inhibitor crizotinib, which would likely not have been identified in our in-house panel testing (Schoch et al. 2020). This emphasizes the value of a combination of whole exome, genome, and transcriptome sequencing in soft tissue sarcomas and could represent a valid alternative to in-house panel NGS testing, which might not sufficiently reflect the diverse biology of BS/STS.

In contrast to other studies, the rate of therapeutic implementation in our cohort is high (31.3% of patients with actionable alterations). In other studies, MTB recommendations were followed through in 3–31.8% of patients with druggable alterations (Rodler et al. 2021; Heinrich et al. 2022; Dorman et al. 2023; Gusho et al. 2022; Sultova et al. 2021; Horak et al. 2021). Compared to the overall cohort, the ten patients treated according to their MTB recommendation were younger (mean 38 vs. 53 years) possibly indicating a selection bias toward patients with a good performance status. Two patients received the MTB-based therapy in addition to conventional chemotherapy, emphasizing the value of personalized combination therapies to combat drug resistance. With seven of ten patients (70%) benefiting from MTB-based therapy in terms of disease stabilization for more than three months and six of eight patients (75%) deriving a clinical benefit defined as a PFSr > 1.3, our results should motivate for routine use of NGS testing for sarcoma patients. In comparison, Horak et al. demonstrated a PFSr > 1.3 under treatment with molecularly targeted therapies in 35% of soft tissue sarcoma patients, and specific histological subtypes such as synovial sarcoma or GIST (PFSr > 1.3 in 50% of patients, respectively) were among the entities associated with the most significant therapeutic benefits. In their study, cases with exceptional clinical benefit were often associated with detection of RNA-based biomarkers and treatment with TKIs. In contrast, targeted therapies appeared to be mostly ineffective in bone sarcoma patients (PFSr > 1.3 in 6% of patients) (Horak et al. 2021). Due to the low sample size in our analysis, possible selection bias and variety of histological subtypes, it is currently difficult to draw conclusions on our high rate of therapeutic changes and benefits. While several studies suggest that NGS has a significant impact in aiding diagnosis and selecting targeted therapies in sarcomas, clinical trials such as the MULTISARC trial (NCT03784014) are needed to determine the final role of comprehensive genomic profiling in sarcomas.

One of our main findings is the latency between initial diagnosis and NGS analysis: the median time from initial diagnosis to MTB discussion was 18.7 months (range 0.3–283.2 months). For patients with synchronous metastatic disease, this time interval was 7.6 months (range 0.3–85.8 months). 84.8% of patients demonstrated metastatic disease at initiation of CGP compared to 43.5% of patients at initial diagnosis. In contrast, Rodler et al. observed that genitourinary cancer patients were mainly included at primary presentation with metastatic disease (Rodler et al. 2021). In pancreatic cancer, a cancer with a notoriously bad prognosis, Dorman et al. observed a median time of 5.5 months between initial diagnosis and discussion in the MTB, owing to the fact that most patients with metastatic pancreatic cancer undergo tumor profiling at initial presentation (Dorman et al. 2023). Regarding the dismal prognosis of BS/STS patients with metastatic disease, the long interval between initial diagnosis and discussion in the MTB raises the question whether comprehensive genomic profiling will positively impact the outcome of patients at later stages of their respective disease. In fact, it has been shown that most patients benefit from targeted agents rather early in the course of their disease (Subbiah and Kurzrock 2018; Westin and Kurzrock 2012). A growing number of molecularly guided clinical (basket) trials are being conducted across the globe, often with limited treatment slots available in a given disease. As such, early and broad integration of biomarker testing helps to identify patients that could potentially benefit from innovative treatments within a clinical trial (Mateo et al. 2022). This assumption is supported by the fact that patients with dedifferentiated liposarcoma characteristically carry MDM2 (and CDK4/6) amplifications (Binh et al. 2005) and novel MDM2 inhibitors are tested in the first-line setting (NCT05218499).

Limitations of our study are the relatively small and very heterogeneous cohort of patients, which remains a typical finding in rare cancers such as bone and soft tissue sarcomas. Our study however reflects the experience of one of the largest sarcoma centers in Germany and Europe. To improve evidence for the routine use of NGS, a structured registry and follow-up program has been initiated at our institution which will allow further evaluation of the benefit of the CCC^LMU^ Munich molecular tumor board (Heinrich et al. 2022).

## Conclusion

With comprehensive genomic profiling becoming a cornerstone in the management of patients with advanced malignancies and the growing number of molecularly guided treatment options, we anticipate that an increasing number of patients with bone and soft tissue sarcomas will derive benefit from the concept of precision oncology. In fact, our experience shows that a significant number of patients with bone and soft tissue sarcomas benefit from targeted treatment options when treated at a large-volume center and in the setting of a dedicated precision oncology program.

## Data Availability

The data presented in this study are available on specific request from the corresponding author. The data are not publicly available for reasons of data protection and data economy.

## References

[CR1] Amary MF, Bacsi K, Maggiani F, Damato S, Halai D, Berisha F, Pollock R (2011). IDH1 and IDH2 mutations are frequent events in central chondrosarcoma and central and periosteal chondromas but not in other mesenchymal tumours. J Pathol.

[CR2] Andrikopoulou A, Terpos E, Chatzinikolaou S, Apostolidou K, Ntanasis-Stathopoulos I, Gavriatopoulou M, Dimopoulos MA, Zagouri F (2021). Tp53 mutations determined by targeted Ngs in breast cancer: a case-control study. Oncotarget.

[CR3] Beckingsale TB, Shaw C (2017). Epidemiology of bone and soft-tissue sarcomas. Orthopaedics and Trauma.

[CR4] Binh MB, Nguyen X-G, Guillou L, de Pinieux G, Terrier P, Lagacé R, Aurias A, Hostein I, Coindre JM (2005). MDM2 and CDK4 immunostainings are useful adjuncts in diagnosing well-differentiated and dedifferentiated liposarcoma subtypes. Am J Surg Pathol.

[CR5] Brennan B, Laura K, Perrine M-B, Javier M-B, Hans G, Nathalie G, Sandra JS (2020). Comparison of two chemotherapy regimens in Ewing Sarcoma (ES): overall and subgroup results of the Euro Ewing 2012 randomized trial (EE2012). J Clin Oncol.

[CR6] Chakravarty D, Johnson A, Sklar J, Lindeman NI, Moore K, Ganesan S, Lovly CM (2022). Somatic genomic testing in patients with metastatic or advanced cancer: ASCO provisional clinical opinion. J Clin Oncol.

[CR7] Cote GM, He J, Choy E (2018). Next-generation sequencing for patients with sarcoma: a single center experience. Oncologist.

[CR8] DeMatteo RP, Ballman KV, Antonescu CR, Maki RG, Pisters PWT, Demetri GD, Blackstein ME (2009). Adjuvant imatinib mesylate after resection of localised, primary gastrointestinal stromal tumour: a randomised, double-blind, placebo-controlled trial. Lancet.

[CR9] Dorman K, Zhang D, Heinrich K, Reeh L, Weiss L, Haas M, Beyer G (2023). Precision oncology in pancreatic cancer: experiences and challenges of the CCC Munich LMU molecular tumor board. Target Oncol.

[CR10] Ferrari S, Stefan SB, Sigbjørn S, Alessandra L, Gerlinde E, Kirsten SH, Davide D (2018). EURO-BOSS: a European study on chemotherapy in bone-sarcoma patients aged over 40: outcome in primary high-grade osteosarcoma. Tumori.

[CR11] Gennari A, André F, Barrios CH, Cortés J, De Azambuja E, Demichele A, Dent R, Fenlon D, Gligorov J, Hurvitz SA (2021). ESMO clinical practice guideline for the diagnosis, staging and treatment of patients with metastatic breast cancer 5 behalf of the ESMO guidelines committee. Ann Oncol.

[CR12] Glabbeke MV, van Oosterom AT, Oosterhuis JW, Mouridsen H, Crowther D, Somers R, Verweij J, Santoro A, Buesa J, Tursz T (1999). Prognostic factors for the outcome of chemotherapy in advanced soft tissue sarcoma: an analysis of 2,185 patients treated with anthracycline-containing first-line regimens–a European organization for research and treatment of cancer soft tissue and bone. J Clin Oncol.

[CR13] Gronchi A, Palmerini E, Quagliuolo V, Broto JM, Pousa AL, Grignani G, Brunello A (2020). Neoadjuvant chemotherapy in high-risk soft tissue sarcomas: final results of a randomized trial from Italian (ISG), Spanish (GEIS), French (FSG), and Polish (PSG) sarcoma groups. J Clin Oncol.

[CR14] Gronchi A, Miah AB, Dei Tos AP, Abecassis N, Bajpai J, Bauer S, Biagini R (2021). Soft tissue and visceral sarcomas: ESMO–EURACAN–GENTURIS clinical practice guidelines for diagnosis, treatment and follow-up. Ann Oncol.

[CR15] Gusho CA, Weiss MC, Lee L, Gitelis S, Blank AT, Wang D, Batus M (2022). The clinical utility of next-generation sequencing for bone and soft tissue sarcoma. Acta Oncol.

[CR16] Heinrich K, Miller-Phillips L, Ziemann F, Hasselmann K, Rühlmann K, Flach M, Biro D (2022). Lessons learned: the first consecutive 1000 patients of the CCC Munich LMU molecular tumor board. J Cancer Res Clin Oncol.

[CR17] Hoff DD, Von JJ, Stephenson PR, Loesch DM, Borad MJ, Anthony S, Jameson G (2010). Pilot study using molecular profiling of patients’ tumors to find potential targets and select treatments for their refractory cancers. J Clin Oncol.

[CR18] Horak P, Klink B, Heining C, Gröschel S, Hutter B, Fröhlich M, Uhrig S (2017). Precision oncology based on omics data: the NCT Heidelberg experience. Int J Cancer.

[CR19] Horak P, Heining C, Kreutzfeldt S, Hutter B, Mock A, Hüllein J, Fröhlich M (2021). Comprehensive genomic and transcriptomic analysis for guiding therapeutic decisions in patients with rare cancers. Cancer Discov.

[CR20] Hu J, Cao J, Topatana W, Juengpanich S, Li S, Zhang B, Shen J, Cai L, Cai X, Chen M (2021). Targeting mutant P53 for cancer therapy: direct and indirect strategies. J Hematol Oncol.

[CR21] Huemer F, Thaler J, Piringer G, Hackl H, Pleyer L, Hufnagl C, Weiss L, Greil R (2018). Sidedness and TP53 mutations impact OS in anti-EGFR but not anti-VEGF treated MCRC: an analysis of the KRAS Registry of the AGMT (Arbeitsgemeinschaft Medikamentöse Tumortherapie). BMC Cancer.

[CR22] Italiano A, Mathoulin-Pelissier S, Le Cesne A, Terrier P, Bonvalot S, Collin F, Michels JJ, Blay JY, Coindre JM, Bui B (2011). Trends in survival for patients with metastatic soft-tissue sarcoma. Cancer.

[CR23] Katz D, Palmerini E, Pollack SM (2018). More than 50 subtypes of soft tissue sarcoma: paving the path for histology-driven treatments. Am Soc Clin Oncol Educ Book.

[CR24] Lochner J, Menge F, Vassos N, Hohenberger P, Kasper B (2020). Prognosis of patients with metastatic soft tissue sarcoma: advances in recent years. Oncol Res Treat.

[CR25] Longley DB, Johnston PG (2005). Molecular mechanisms of drug resistance. J Pathol.

[CR26] Lucchesi C, Emmanuel K, Yecan L, Isabelle S, Simone M-P, Christine C, Antoine I (2018). Targetable alterations in adult patients with soft-tissue sarcomas: insights for personalized therapy. JAMA Oncol.

[CR27] Marabelle A, Fakih MG, Lopez J, Shah M, Shapira-Frommer R, Nakagawa K, Chung HC (2019). Association of tumour mutational burden with outcomes in patients with select advanced solid tumours treated with pembrolizumab in KEYNOTE-158. Ann Oncol.

[CR28] Marko TA, Diessner BJ, Spector LG (2016). Prevalence of metastasis at diagnosis of osteosarcoma: an international comparison. Pediatr Blood Cancer.

[CR29] Massard C, Michiels S, Ferté C, Deley MCL, Lacroix L, Hollebecque A, Verlingue L (2017). High-throughput genomics and clinical outcome in hard-to-treat advanced cancers: results of the MOSCATO 01 trial. Cancer Discov.

[CR30] Mateo J, Steuten L, Aftimos P, André F, Davies M, Garralda E, Geissler J (2022). Delivering precision oncology to patients with cancer. Nat Med.

[CR31] Mosele F, Remon J, Mateo J, Westphalen CB, Barlesi F, Lolkema MP, Normanno N (2020). Recommendations for the use of next-generation sequencing (NGS) for patients with metastatic cancers: a report from the ESMO precision medicine working group. Ann Oncol.

[CR32] Planchard D, Popat S, Kerr K, Novello S, Smit EF, Faivre-Finn C, Mok TS (2018). Metastatic non-small cell lung cancer: ESMO clinical practice guidelines for diagnosis, treatment and follow-up. Ann Oncol.

[CR33] Rodler S, Jung A, Greif PA, Rühlmann K, Apfelbeck M, Tamalunas A, Kretschmer A (2021). Routine application of next-generation sequencing testing in uro-oncology—are we ready for the next step of personalised medicine?. Eur J Cancer.

[CR34] Schoch K, Queenie KGT, Nicholas S, Kristen LD, Allyn M-R, Marie TM, David BG, Yong HJ, Vandana S (2020). Alternative transcripts in variant interpretation: the potential for missed diagnoses and misdiagnoses. Genet Med.

[CR35] Smeland S, Bielack SS, Whelan J, Bernstein M, Hogendoorn P, Krailo MD, Gorlick R (2019). Survival and prognosis with osteosarcoma: outcomes in more than 2000 patients in the EURAMOS-1 (European and American Osteosarcoma Study) cohort. Eur J Cancer.

[CR36] Sosman JA, Kevin BK, Lynn S, Rene G, Anna CP, Jeffrey SW, Grant AM (2012). Survival in BRAF V600-mutant advanced melanoma treated with vemurafenib. N Engl J Med.

[CR37] Stacchiotti S, Frezza AM, Blay JY, Baldini EH, Bonvalot S, Bovée JVMG, Callegaro D (2021). Ultra-rare sarcomas: a consensus paper from the connective tissue oncology society community of experts on the incidence threshold and the list of entities. Cancer.

[CR38] Strauss SJ, Frezza AM, Abecassis N, Bajpai J, Bauer S, Biagini R, Bielack S (2021). Bone sarcomas: ESMO–EURACAN–GENTURIS–ERN PaedCan clinical practice guideline for diagnosis, treatment and follow-up. Ann Oncol.

[CR39] Subbiah V, Kurzrock R (2018). Challenging standard-of-care paradigms in the precision oncology era. Trends in Cancer.

[CR40] Sultova E, Benedikt Westphalen C, Jung A, Kumbrink J, Kirchner T, Mayr D, Rudelius M (2021). Implementation of precision oncology for patients with metastatic breast cancer in an interdisciplinary Mtb setting. Diagnostics.

[CR41] Tannock IF, Hickman JA (2019). Editorials molecular screening to select therapy for advanced cancer?. Ann Oncol.

[CR42] Tap WD, Villalobos VM, Cote GM, Burris H, Janku F, Mir O, Beeram M (2020). Phase I study of the mutant IDH1 inhibitor ivosidenib: safety and clinical activity in patients with advanced chondrosarcoma. J Clin Oncol.

[CR43] Tirode F, Surdez D, Ma X, Parker M, Deley MCL, Bahrami A, Zhang Z (2014). Genomic landscape of Ewing sarcoma defines an aggressive subtype with co-association of STAG2 and TP53 mutations. Cancer Discov.

[CR44] Van Cutsem E, Cervantes A, Adam R, Sobrero A, Van Krieken JH, Aderka D, Aranda Aguilar E (2016). ESMO consensus guidelines for the management of patients with metastatic colorectal cancer. Ann Oncol.

[CR45] Westin JR, Kurzrock R (2012). It’s about time: lessons for solid tumors from chronic myelogenous leukemia therapy. Mol Cancer Ther.

[CR46] Yarchoan M, Hopkins A, Jaffee EM (2017). Tumor mutational burden and response rate to PD-1 inhibition. N Engl J Med.

[CR47] Yarchoan M, Albacker LA, Hopkins AC, Montesion M, Murugesan K, Vithayathil TT, Zaidi N (2019). PD-L1 expression and tumor mutational burden are independent biomarkers in most cancers. JCI Insight.

